# Case report from a pediatric multidrug-resistant tuberculosis case with Tsukamurella co-infection identified by whole-genome sequencing: a diagnostic and therapeutic challenge

**DOI:** 10.1186/s12879-026-13524-y

**Published:** 2026-05-20

**Authors:** Yeshiwork Abebaw, Arash Ghodousi, Dawit Hailu Alemayehu, Abaysew Ayele, Gebremedhin Gebremicael, Yohannes Nugusa, Genet Asaye, Getu Diriba, Muluwork Getahun, Andrea MaurizioCabibbe, Markos Abebe, Anandi Sheth, Rahel Argaw, Woldaregay Erku Abegaz

**Affiliations:** 1 Department of Microbiology, Immunology, and Parasitology, College of Health Sciences, Addis Ababa University, Addis Ababa, Ethiopia; 2 Ethiopian Public Health Institute, Addis Ababa, Ethiopia; 3 Vita-Salute San Raffaele University, Milan, Italy; 4 Emerging Bacterial Pathogens Unit, IRCCS San Raffaele Scientific Institute, Milan, Italy; 5 Armauer Hansen Research Institute, Addis Ababa, Ethiopia; 6 Selale University Comprehensive Specialized Hospital, Fitch, Ethiopia; 7 Ministry of Health, Addis Ababa, Ethiopia; 8 Department of Medicine, Emory University School of Medicine, Atlanta, United States of America; 9 Department of Pediatrics and Child Health, College of Health Sciences, Addis Ababa University, Addis Ababa, Ethiopia

**Keywords:** Tsukamurella species, Tuberculosis, Mycobacterium tuberculosis-Tsukamurella species co-infection, Whole genome sequencing

## Abstract

**Background:**

Tsukamurella species are rarely reported pathogens that can clinically mimic tuberculosis (TB). We report a challenging case of Tsukamurella species co-occurring with multidrug-resistant tuberculosis (MDR-TB) in a pediatric patient.

**Case presentation:**

A 12-year-old HIV-negative female with a previous history of tuberculosis was admitted in August 2021 to Selale University Comprehensive Specialized Hospital in Ethiopia. She was diagnosed with MDR-TB and started on an all-oral bedaquiline-containing regimen. Whole genome sequencing later identified co-infection with Mycobacterium tuberculosis Lineage 3 (Central Asian Strain) and Tsukamurella spp.

**Conclusion:**

This case highlights the critical importance of considering Tsukamurella sp. co-infection in patients with culture-positive MDR-TB who exhibit an atypical or persistent clinical course. The diagnostic mimicry between these pathogens necessitates the use of advanced microbiological techniques for accurate identification, as standard TB workflows may overlook such rare co-infections.

**Supplementary Information:**

The online version contains supplementary material available at 10.1186/s12879-026-13524-y.

## Introduction

Tuberculosis (TB) remains a major global health threat, a challenge significantly compounded by the rising tide of drug-resistant strains, particularly in low- and middle-income countries [1]. The burden is starkly evident in the pediatric population; recent estimates indicate a rise in childhood TB cases just in a single year period from 1.25 million in 2023 to 1.3 million in 2024 [2]. The burden of TB is exacerbated by co-infections such as human immunodeficiency virus (HIV) [3]. TB–HIV co-infection in children is associated with well-documented diagnostic delays, complex clinical management, and poor treatment outcomes [3–4].

Beyond HIV, immunocompromised individuals are also vulnerable to a spectrum of rare opportunistic pathogens that can mimic TB, creating diagnostic confusion, and one of such pathogens being *Tsukamurella* [5–6]. First described in 1977, the genus *Tsukamurella* now comprises approximately 14 species [6]. The clinical significance of *Tsukamurella* stems from its phylogenetic and morphological similarity to *Mycobacterium* and *Nocardia* species. Like MTBC, *Tsukamurella* are aerobic, weakly acid-fast, Gram-positive bacilli that can grow in standard mycobacterial culture media, potentially leading to misidentification or delayed diagnosis if molecular confirmation is not pursued [5–7].

Currently, the understanding of this pathogen is largely confined to case reports, with no standardized diagnostic or therapeutic guidelines [7].

We present a case that encapsulates these overlapping challenges: a *Tsukamurella* species co-infected with genomically identified multidrug-resistant TB (MDR-TB) in a pediatric patient. This report highlights the critical need for advanced diagnostic techniques to differentiate such complex co-infections and to guide effective therapy in high TB-burden settings.

## Case presentation

A 12-year-old, HIV-negative female was admitted to Selale University Comprehensive Specialized Hospital, Fitche, Ethiopia, with a four-week history of fever, night sweats, productive cough, pleuritic chest pain, and progressive shortness of breath. Her symptoms began eight weeks after self-discontinuing her first-line anti-tuberculosis treatment. The patient had initially completed 12 weeks of therapy but subsequently missed all follow-up appointments. The contributing factors were the patient’s subjective feeling of recovery, her father’s belief that she was cured, and the considerable distance to the healthcare facility. She had no known drug allergies, nor was any direction given by a clinician to stop the treatment.

On physical examination, the patient was ill-appearing and cachectic, with a body mass index (BMI) of 14.5, placing her in the severely underweight range according to CDC Growth Charts [8]. Vital signs were significant for tachypnea (24 breaths/minute) and profound hypoxia, with an oxygen saturation of 84% on room air. Her temperature was 36.9 °C, and her pulse was 82 beats/minute. Auscultation of the chest revealed diffusely decreased breath sounds and rales. The patient also reported episodes of hemoptysis.

**Fig. 1:**
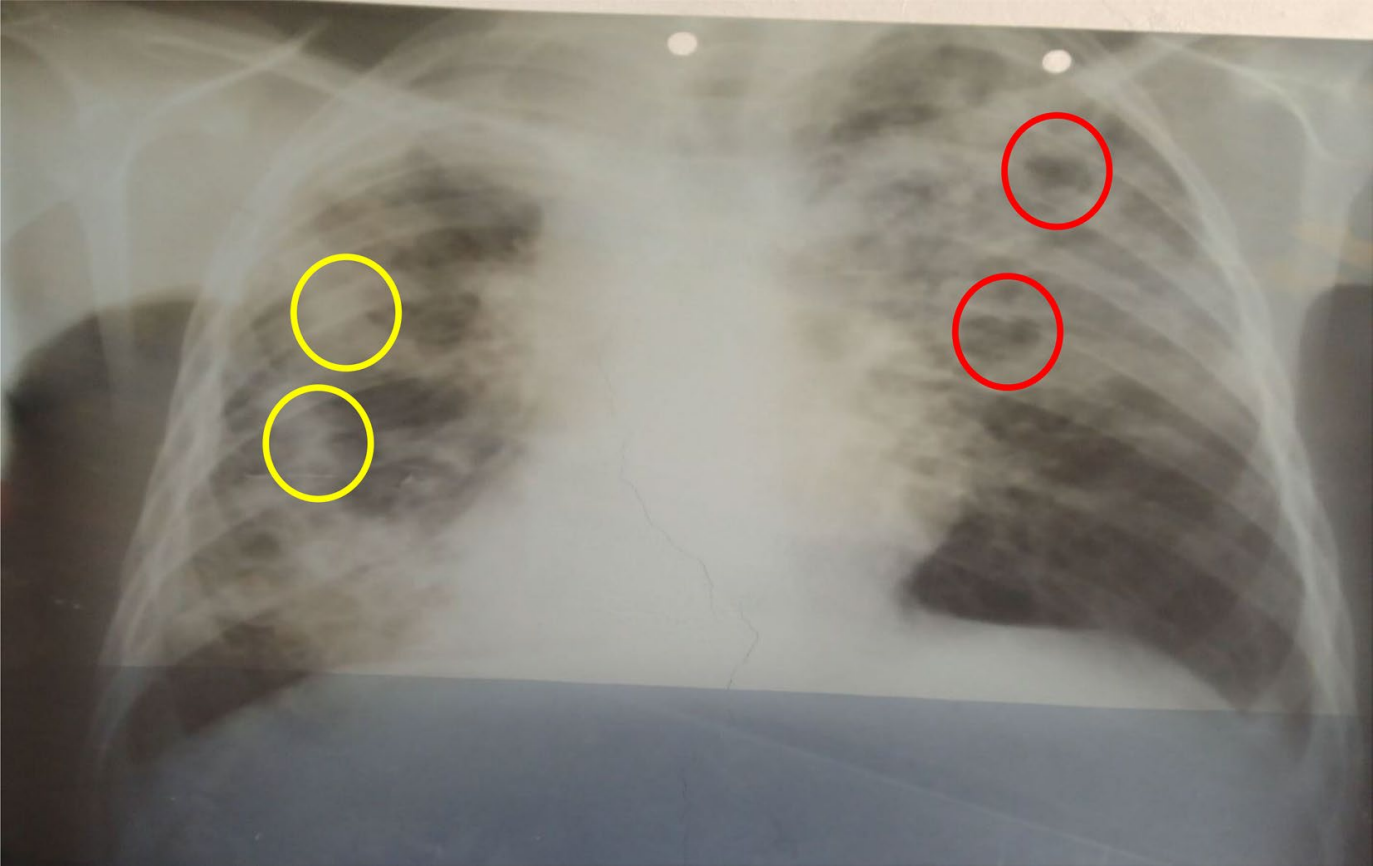
Chest X-ray of a patient diagnosed with advanced bilateral pulmonary tuberculosis Yellow indicates consolidation, and red indicates cavitation

Baseline laboratory investigations, including liver enzymes, Chemistry for GPT, Creatinine, Urea, Potassium, Albumin, Sodium, Chloride, and Hemoglobin, were tested, and all results were within normal ranges. Moreover, a chest X-ray examination was performed, revealing multiple cavitation formations, opacification, and consolidation in the lungs, as shown in Fig. 1.

### Microbiological investigations

**Fig. 2:**
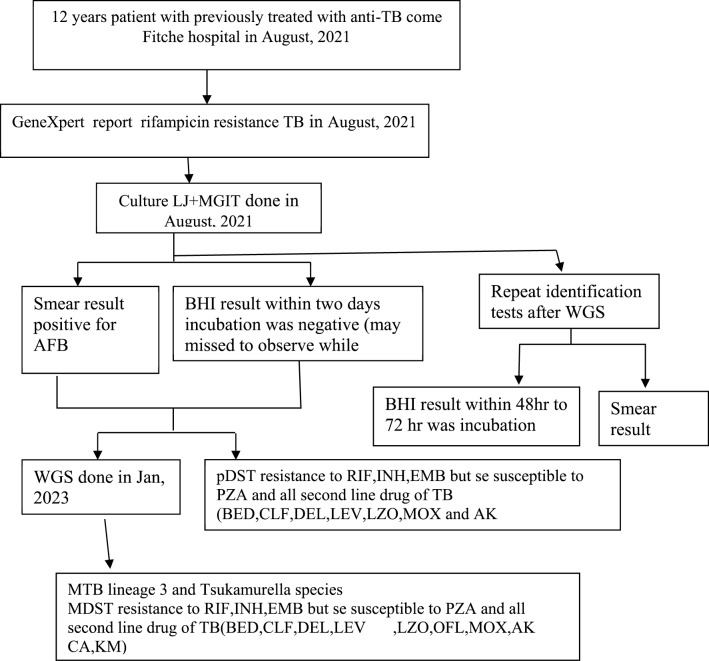
Laboratory diagnosis work flow. Abbreviation: Isoniazid, INH; Rifampicin, RIF; Levofloxacin, LEV; Moxifloxacin, MOX; Amikacin, AK; Capreomycin, CA; Kanamycin, KM; Ethionamide, ETH; Pyrazinamide, PZA; Bedaquiline, BED; Clofazimine, CLF; Delamanid, DEL; Ethambutol, EMB; Linezolid, LOZ, Whole-Genome Sequencing, WGS

A sputum sample was subjected to the GeneXpert MTB/RIF assay, which was positive for *Mycobacterium tuberculosis* complex (MTBC) and rifampicin resistance. Based on this result, a presumptive diagnosis of MDR-TB was made, and an additional sputum sample was sent to the National Tuberculosis Reference Laboratory for TB-culture and phenotypic drug susceptibility testing (DST) for first- and second-line agents at WHO-recommended critical concentrations [9–10] as indicated in the laboratory work flow (Fig. 2).

The sputum sample was cultured using the Mycobacteria Growth Indicator Tube (MGIT). Growth was detected, and because MGIT supports the growth of various mycobacteria, the sample was subjected to Ziehl-Neelsen staining, which revealed acid-fast bacilli (AFB). To rule out contamination, the culture was simultaneously inoculated onto Brain Heart Infusion (BHI) agar. After 48 h of incubation, the BHI agar showed no growth (which may be missed or not detected at this stage because of low organism load or because of the nature of the bacteria since it is slow growing bacteria), confirming the absence of contaminating bacteria, while the AFB smear remained positive. The mycobacterial isolate was subsequently confirmed as *M. tuberculosis* complex using a rapid immunochromatographic assay (Capilia TB-Neo Test).

Phenotypic DST results confirmed MDR-TB, demonstrating resistance to isoniazid, rifampicin, and ethambitol but susceptibility to pyrazinamide, as well as to the second-line agents bedaquiline, clofazimine, delamanid, levofloxacin, linezolid, and moxifloxacin.

### Phylogenetic analysis confirms the isolate’s identity as *Tsukamurella*

**Fig. 3:**
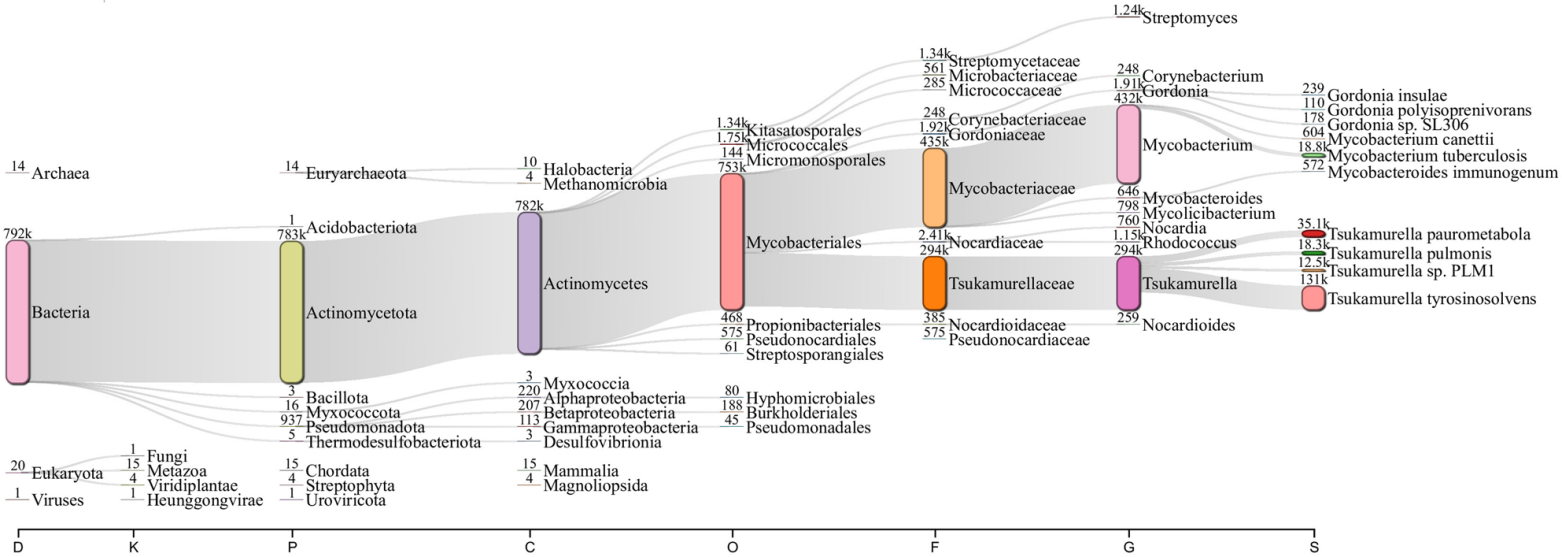
Sankey diagram of whole genome sequencing data. The visualization demonstrates a co-dominance of two primary genera within the phylum Actinomycota which M. tuberculosis (648 reads) with low abundant compeered Tsukamurella species which highly abundant, with T. paurometabola (35,000 and T. pulmonis (18,800 reads)

As part of a separate research study on childhood TB, the isolate underwent whole-genome sequencing (WGS) as described previously [11]. The DNA was extracted from heat-inactivated MGIT isolates as described in a previously published protocol using the Cetyltrimethylammonium Bromide-lysozyme (CTAB) method [12], and Whole-genome sequencing was performed using the Illumina NextSeq 500/550 platform. Quality control included adapter trimming and removal of low-quality reads, which were removed through trimming and filtering using *Trimmomatic* (v0.33) [13]. Filtered reads were analyzed with Kraken 2 for taxonomic profiling, a tool that assigns taxonomic labels based on k-mer comparisons against a reference database [14, 15]. This tool provided an initial profile of the microbial composition, indicating the presence of multiple organisms. To confirm species identity, reads were mapped to reference genomes of Mycobacterium tuberculosis H37Rv [16]. Mapping quality metrics were evaluated with Samtools stats, where the average depth of coverage was 15× for MTBC and 12× for *Tsukamurella* [17]. The taxonomic abundance data generated by Kraken 2 were then visualized using a Sankey diagram, which effectively illustrates the proportional flow and relationship of sequence reads from the domain level down to the species level. This visualization played a crucial role in confirming the co-dominance of MTBC and *Tsukamurella* species within the clinical sample, providing a clear and quantitative representation of the co-infection (Fig. 3). Sequencing analysis confirmed the presence of MTBC species, including *M. tuberculosis* (648 reads), *M. canettii* (183 reads), and *M. immunogenum* (122 reads), although these were present at very low abundance. In contrast, *Tsukamurella* species were highly abundant, with *T. paurometabola* (35,000) and *T. pulmonis* (18,800 reads) dominating the microbial profile, while *T. tyrosinosolvens* (259 reads) was minor.

In addition, the MTBC sequencing data were analyzed by TB-profiler as described previously [11]. Variant calling confirmed the presence of mutations associated with drug resistance, specifically *rpoB* Ser450Leu (p.Ser450Leu) for rifampicin resistance, *katG* Ser315Thr (p.Ser315Thr) for isoniazid resistance, and *embB* (p.Asp354Ala) for Ethambutol, and the MTB lineage that co-occurred with *Tsukamurella* spp. was identified as Lineage 3.

**Fig. 4:**
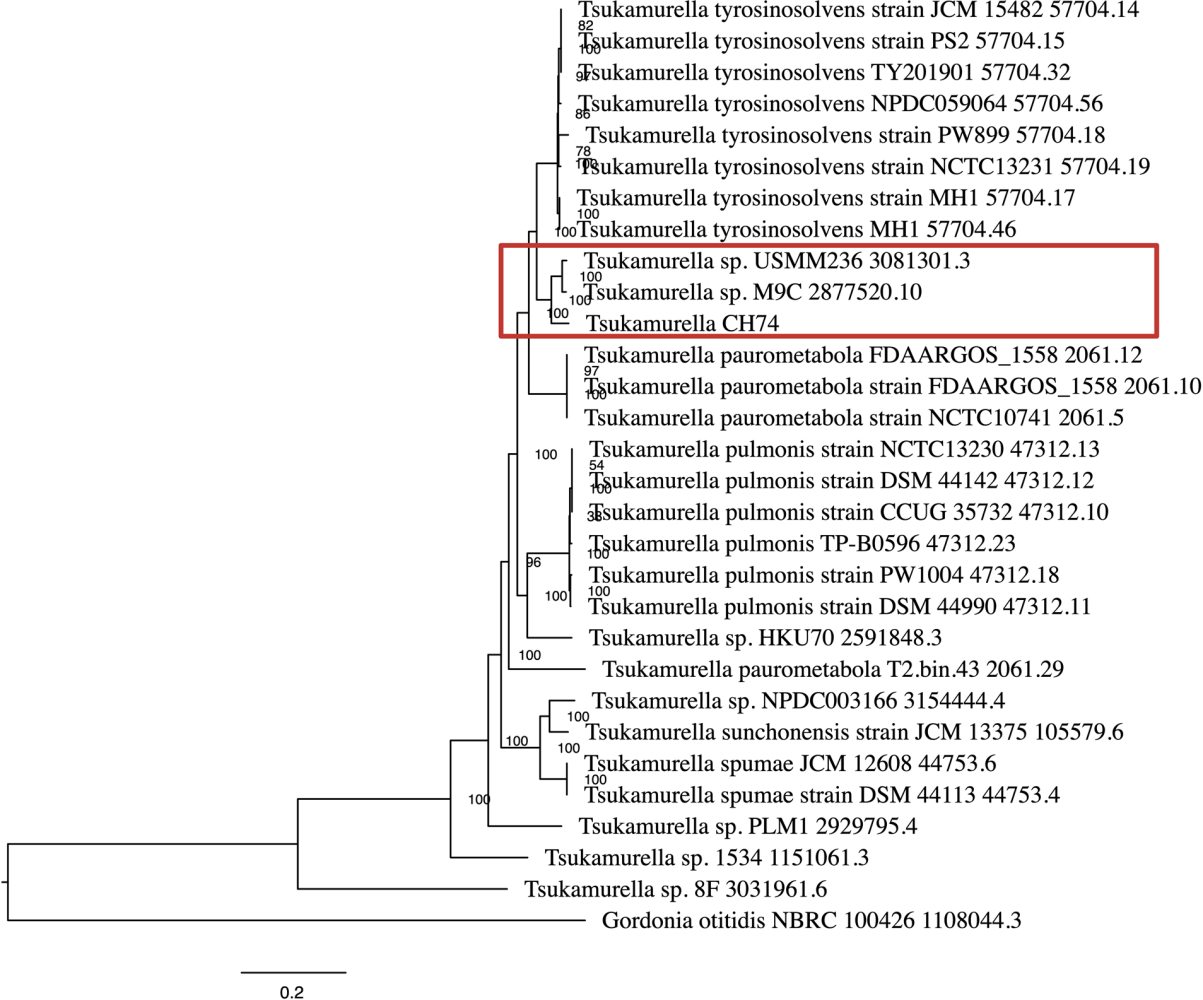
Maximum-likelihood phylogenetic tree of the Tsukamurella genus. The tree illustrates the evolutionary placement of the clinical isolate CH74 among reference Tsukamsurella species. The tree was constructed from whole-genome sequences and rooted using Gordonia otitidis NBRC 100,426 as an outgroup. The clustering of CH74 with Tsukamurella sp. M9C and Tsukamurella sp. USMM236 with high bootstrap support confirms its taxonomic classification within the genus.Accession numbers of reference genomes in supplement 1

To definitively classify the clinical isolate CH74 and elucidate its evolutionary relationships, a maximum-likelihood phylogenetic tree was constructed using whole-genome sequences, taking representative *Tsukamurella* species as references and *Gordonia otitidis* as the outgroup (Fig. 3, supplement 1). The analysis confidently placed isolate CH74 within the *Tsukamurella* genus. As shown in Fig. 4, CH74 forms a distinct, well-supported clade with the environmental strains *Tsukamurella* sp. M9C and *Tsukamurella* sp. USMM236 (bootstrap value = 98%). This robust clustering confirms the accurate taxonomic assignment of our clinical isolate and suggests a close evolutionary relationship between these strains, which may share a recent common ancestor. The tree clearly differentiates CH74 from other established pathogenic species within the genus, such as *Tsukamurella paurometabola* and *Tsukamurella pulmonis*.

After the sequence data became known, the initial sample was inoculated to see the presence of bacteria other than MTBC. Subsequently, well-defined colonies grew on BHI agar within 48–72 h, a growth rate atypical for MTBC strains. A subsequent AFB smear of these colonies was positive, confirming that the acid-fast bacilli initially observed were a mixture of MTBC and the rapidly growing *Tsukamurella* species, which had been overlooked in the primary culture workflow.

The patient was treated with a nine-month all-oral, bedaquiline-containing regimen before WGS result was known since the case identified accidently during sequencing of MTBC isolates for research purpose. The regimen includes Bedaquiline, Levofloxacin, Clofazimine, Ethambutol, Pyrazinamide, and High-dose isoniazid for MDR-TB, and reported as cured, with no adverse events. Her BMI increased from 14.5 to 16.4. Culture and smear results were negative. Despite the co-infection with *Tsukamurella* spp., no additional specific treatment for *Tsukamurella* was administered, as it was not known at the time of treatment initiation. However, follow-up of the patient confirmed that she remained relapse-free two years after completing the treatment, with no recurrence of symptoms or infection, indicating successful resolution of both the co-infection.

## Discussion

*Tsukamurella* species are rare but emerging pathogens that can affect both children and adults, including infants [18]. Most of the available evidence is derived from case reports, which are frequently reported as catheter-related bloodstream infections and soft tissue infections in immunocompromised hosts [5, 6]. Moreover, *Tsukamurella* species have also been isolated from pulmonary infections as pneumonia [5, 6]. Due to overlapping clinical and microbiological characteristics with *Mycobacterium tuberculosis*, pulmonary *Tsukamurella* infections are often misidentified as tuberculosis, which may lead to misdiagnosis and inappropriate treatment. Limited studies showed that co-infection of *Tsukamurella* and *Mycobacterium tuberculosis* confections [19].

Our finding also confirmed the simultaneous occurrence of the two pathogens in a female child after initially overlooking the possibility of any co-infection, highlight the need for accurate diagnostic approaches to differentiate *Tsukamurella* from MTBC, particularly in high TB-burden settings. To our knowledge, no previous report has described genomically confirmed *Tsukamurella* and MDR-TB co-infection in a pediatric patient in Ethiopia, representing a significant contribution to the limited literature on rare mycobacterial-like pathogens in children in high TB burden countries. The findings highlight that conventional diagnostic tools, such as smear microscopy and culture alone, can lead to misdiagnosis, particularly in cases involving atypical pathogens. In this case, the use of whole-genome sequencing was pivotal in identifying the co-infecting *Tsukamurella* species, reinforcing the indispensable role of advanced molecular diagnostics in resolving ambiguous or atypical clinical presentations [20]. Whole genome sequencing not only enabled accurate species-level identification but also provided insights into potential resistance determinants, which are otherwise difficult to ascertain due to the lack of standardized susceptibility testing protocols for *Tsukamurella* [7].

Previous studies showed that patient with Tsukamurella can be treated and cured with regiment with levofloxacin, sulfamethoxazole, or trimethoprim [21], or with ciprofloxacin and clarithromycin [22]. Study also showed that *Tsukamurella* is susceptible to amikacin, ciprofloxacin, imipenem, doxycycline, linezolid, and sulfamethoxazole [23]. In our case, the patient’s successful outcome on a bedaquiline-based MDR-TB regimen, which include Levofloxacin and clofazimine with sustained remission over a two-year follow-up, suggests that the treatment may have been effective against both *M. tuberculosis* and *Tsukamurella* species. However, this result should be interpreted with caution. *Tsukamurella* species are known to exhibit intrinsic and variable resistance to many anti-TB drugs [21, 24], and the lack of validated susceptibility testing for this genus means optimal therapy remains uncertain. This case also raises broader implications for TB-endemic settings, where diagnostic infrastructure may be limited, and co-infections with rare or atypical organisms may go undetected. Clinicians and microbiologists should maintain a high index of suspicion for dual infections, particularly in patients with atypical clinical courses, poor treatment response, or unusual microbiological findings. Strengthening laboratory capacity for molecular diagnostics, including WGS, is essential for improving pathogen identification and guiding appropriate management.

A key limitation of this case is the absence of a dedicated drug susceptibility profile for the *Tsukamurella* isolate, which restricts our ability to draw firm conclusions about drug efficacy. Moreover, Whole-genome sequencing was performed on the initial diagnostic isolate. Follow-up sputum samples collected during MDR-TB treatment were negative. Therefore, we were unable to confirm persistent infection or repeated isolation of *Tsukamurella*. Conversely, a major strength is the long-term follow-up, which confirms a durable clinical cure, lending support to the effectiveness of the chosen regimen.

## Conclusion

In conclusion, this report presents a rare, genomically confirmed case of *Tsukamurella* spp. Co-occurring with MDR-TB in a pediatric patient. The case highlights a critical diagnostic challenge: the pathological similarities between *Tsukamurella* and *M. tuberculosis* can lead to the misattribution of treatment failure solely to MDR-TB, potentially overlooking a consequential co-infection. Therefore, in patients with a suspected TB relapse or poor treatment response, clinicians should consider the possibility of a *Tsukamurella* co-infection. Where available, confirmation requires molecular methods such as secA1 or 16 S rRNA gene sequencing, or MALDI-TOF MS, which are essential for accurate differentiation from mycobacteria [25].

There is an urgent need for well-designed studies to determine the prevalence of this co-infection and to develop accessible diagnostic tools for resource-constrained settings. Improving detection and understanding of this pathogen will be essential for guiding appropriate treatment and improving patient outcomes.

## Supplementary Information

Below is the link to the electronic supplementary material.

Supplementary Material 1

## Data Availability

The datasets generated and analyzed during the current study are available in publicly accessible repositories. The raw sequencing data were submitted to NCBI as FASTQ files under the study Accession Number: PRJNA1204469.

## Abbreviations

AFB: Acid-fast bacilli
BHI: Brain Heart Infusion
HIV: Human Immunodeficiency Virus
MDR-TB: Multidrug-resistant tuberculosis
MGIT: Mycobacteria Growth Indicator Tube
MTBC: Mycobacterium tuberculosis complex
WGS: Whole-genome Sequencing
